# Maximal aerobic and anaerobic power and time performance in 800 m double poling ergometer

**DOI:** 10.1007/s00421-023-05149-9

**Published:** 2023-02-07

**Authors:** Øyvind Støren, Arnstein Sunde, Jan Helgerud, Jan-Michael Johansen, Lars-Erik Gjerløw, Henrik Hjortland, Eva Maria Støa

**Affiliations:** 1 Department of Sports, Physical Education and Outdoor Studies, University of South-Eastern Norway, Bø, Norway; 2 Department of Natural Sciences and Environmental Health, University of South-Eastern Norway, Bø, Norway; 3 Department of Circulation and Medical Imaging, Norwegian University of Science and Technology, Trondheim, Norway; 4 Myworkout, Medical Rehabilitation Centre, Trondheim, Norway

**Keywords:** Double poling ergometer performance, Aerobic capacity, Anaerobic capacity, Sprint performance, Anaerobic sprint reserve

## Abstract

The purpose of this study was to investigate to what extent aerobic power (MAP), maximal anaerobic power (MANP), anaerobic capacity measured as time to exhaustion at 130% MAP (TTE), and maximal accumulated oxygen deficit (MAOD) correlated with 800 m double poling time trial performance (800TT) in a ski ergometer. A second aim was to investigate the relationship between TTE and MAOD, and to what extent TTE and MAOD would relate to anaerobic power reserve (APR). Eighteen cross-country skiers were tested for peak oxygen uptake (*V*O_2peak_) and oxygen cost of double poling to assess MAP. Peak power measurements during a 100 m TT were performed to assess MANP. TTE and an 800TT with continuous *V*O_2_ measurements were performed to assess time performance and MAOD. All tests were performed on a ski ergometer. Both MAP and MANP correlated strongly (*r* = − 0.936 and − 0.922, respectively, *p* < 0.01) with 800TT. Neither TTE nor MAOD correlated with 800TT. TTE correlated moderately with MAOD, both in mL kg^−1^ and in %*V*O_2peak_ (*r* = 0.559, *p* < 0.05 and 0.621, *p* < 0.01, respectively). Both TTE and MAOD seemed to be a product of APR. These results suggest focusing on MAP and MANP, but not anaerobic capacity to explain time performance in an event with approximately 3 min duration.

**Supplementary Information:**

The online version contains supplementary material available at 10.1007/s00421-023-05149-9.

## Introduction

Middle distance performance such as the sprint event in cross-country skiing or middle-distance running put great demands on both aerobic (~ 80%) and anaerobic (~ 20%) metabolism (Brandon 1995; Losnegard et al. 2012; Andersson et al. 2017; Støren et al. 2021). Accordingly, several studies report that overall sprint performance in cross country skiing is heavily influenced by aerobic contributors such as maximal oxygen uptake (*V*O_2max_) and oxygen cost of skiing (*C*), i.e., maximal aerobic speed (MAS) (Stöggl et al. 2007; Sandbakk et al. 2011b, c; Losnegard et al. 2012; Carlsson et al. 2016; Andersson et al. 2017; Hébert-Losier et al. 2017). Both maximal sprint skiing velocity (MANS) and maximal accumulated oxygen deficit (MAOD) have been reported to impact time results in sprint cross country skiing (Stöggl et al. 2007; Mikkola et al. 2010; Sandbakk et al. 2011b; Losnegard et al. 2012; Carlsson et al. 2016; Hébert-Losier et al. 2017). In middle-distance running, Støren et al. (2021) found that time trial performance in 800 m running was primarily dependent on MAS and MANS. In the Støren et al. (2021) study, no impact from anaerobic capacity measured as time to exhaustion (TTE) at 130% of MAS was found, and it seemed that TTE was merely a product of each runner’s individual anaerobic sprint reserve (ASR), i.e., the difference between MAS and MANS (Støren et al. 2021). Some previous studies have investigated the impact of anaerobic capacity measured as MAOD in middle distance running (Ramsbottom et al. 1994; Craig and Morgan. 1998; Nevill et al. 2008; Billat et al. 2009), with equivocal findings. As suggested in Støren et al. (2021), the term anaerobic capacity could be unclear. In a short sprint, it is the maximal velocity of anaerobic energy release that matters, while in a middle-distance event it is the ability to sustain a certain amount of anaerobic energy release over time. The latter thus represents the product of volume ⋅ time (Medbo et al. 1988). The term anaerobic endurance capacity would perhaps better cover a possible impact of MAOD on middle distance running or sprint cross-country skiing time performance. On the other hand, the term endurance may also be misplaced in relation to anaerobic capacity, as it has been suggested that anaerobic capacity is merely a product of anaerobic sprint reserve (ASR) (Støren et al. 2021).

Losnegard et al. (2012, 2013) suggested that differences in sprint performance among elite skiers are more dependent on anaerobic capacity measured as MAOD, than aerobic capacity, since improved performance in a 1000 m time trial (TT) in elite cross-country skiers were related to an increase in MAOD. Further, Losnegard and Hallén (2014) reported higher MAOD in a 1000 m TT in sprint-specialized skiers compared to distance-specialized skiers. However, the sprint-specialized skiers were slower than distance-specialized skiers in the 1000 m TT despite the higher MAOD. The latter makes it difficult to conclude that MAOD is important for sprint ski performance. In Støren et al. (2021), TTE at 130% MAS was used as an indirect measure of anaerobic capacity in 800 m running. However, MAOD was not measured in that study. It was proposed, but not measured that if two athletes should perform TTE, the runner performing the longest time would have the highest MAOD relative to *V*O_2max_. If so, anaerobic power reserve (APR) relative to maximal aerobic power (MAP) should theoretically be a significant determinant of MAOD, as ASR was to TTE in Støren et al. (2021). To our knowledge, no previous studies have investigated ASR or the equivalent APR in double poling (DP), and the relation to sprint performance.

Cross-country skiing in general is performed using several different techniques. To avoid the possible influence of tactical choice of techniques, there may be a benefit of using a single technique like DP. DP would also allow for the use of a ski ergometer, ensuring similar test conditions. It might be argued that time performance in a ski ergometer do not represent time performance in skiing on snow. Those with the best skiing techniques on snow will not necessarily have the best ski ergometer techniques, and vice versa. Also, a large body mass will not impact the performance negatively in the ski ergometer but may increase friction between ski and snow. On the other hand, to investigate the relationships between aerobic and anaerobic power, anaerobic capacity, APR, and time performance in DP in a ski ergometer do not need to be representative for snow skiing performance to have a relevance. Using MAS and MANS, or the equivalents MAP and maximal anaerobic power (MANP), skiing technique and body mass will be embedded in these terms. For example, a good ski ergometer DP technique will give a higher power output in the sprint, and thus enhance MANP. The good technique will also reduce C, and thus give a better MAP. Also, a large body mass will reduce both *C* and peak oxygen uptake (*V*O_2peak_), if expressed per kg body mass. Since MAP is the product of *V*O_2peak_ divided by *C*, MAP will be independent of body mass (Helgerud et al. 2010; Støren et al. 2021; Johansen et al. 2022).

In cross country sprint skiing as in middle distance running, knowledge about performance determining physiological variables is of great importance to design the most effective training programs. The use of the terms MAS and MANS, or the equivalents MAP and MANP have the advantages that the denotations are the same as in the time performance, i.e., time or watt (*w*). TTE at 130% MAP and MAOD measurements, with the latter performed during an actual time trial could give insights to the relationships between anaerobic capacity and TT performance. By investigating the relationships between these variables and time performance, we can indicate which variables determine middle distance performance the most. These indications may further be an important base for future interventions to investigate the causalities in the relationships. The main purpose of the present study was, therefore, to investigate the correlations between MAP and MANP and 800 m DP time performance in a ski ergometer, and further to investigate the role of anaerobic capacity measured as both TTE at 130% MAP and MAOD on 800 m DP time performance. A second aim of the present study was to investigate the relationship between TTE and MAOD, and to what extent TTE and MAOD would be related to APR.

## Methods

### Subjects

Eighteen (5 women and 13 men) moderate- to well-trained subjects aged 24 ± 5 years participated in this study (Table 1). Their performance level ranged from healthy students and recreational skiers, to top national level skiers. In order to be able to detect potential relationships between physiological variables and time performance, a minimum of heterogeneity in time performance was needed. We, therefore, sought to recruit both competitive athletes as well as recreational ones. The study was approved by the institutional research board at the University of Southeastern Norway, the Norwegian Centre for Research Data (NSD, reg 413787) and conducted in accordance with the Helsinki declaration. All subjects gave their written consent to participate, after having received information about the study.

**Table 1:** Subjects characteristics (*N* = 18)

Age (years)	24 ± 5	(19.2)
BM (kg)	76.3 ± 9.9	(13.0)
Height (cm)	178.1 ± 8.3	(4.7)
800TT (s)	192.2 ± 28.8	(15.0)

Values are mean ± standard deviation, with coefficient of variance in percent in parenthesis

*BM* body mass, *kg* kilograms, *cm* centimeters, *800TT* time results in the 800 m, *s* seconds

### Testing procedures

The subjects were tested over 2 different days with a minimum of 1 day in between, and a maximum of 1 week between test days. According to the laboratory standards, the participants were instructed to do only light training the last 24 h before testing, and no food or nutritious drinks were allowed 1 h before the first test. In between tests, the participants were allowed to eat a light meal of energy-rich food and drinks. *V*O_2peak_, C and TTE were tested on day one. All tests were performed in DP on a ski ergometer (Concept 2 Ski ergometer, Concept2, Vermont, USA). The subjects performed a 15-min submaximal workout before the *V*O_2peak_ test. All *V*O_2_ measurements were performed with the metabolic test system, Jaeger Vyntus CPX (CareFusion, GmbH, Hoechberg, Germany), with a mixing chamber. The subjects started with an outlay speed predicted to represent an intensity of approximately 70% of maximal heart rate (HR_max_). All HR measurements were made by Polar s610 HR monitors (Polar Oy, Kempele, Finland). Every 30 s the speed was increased by 0:05 min·500 m^−1^. After 5–7 increases, the subjects were told to pole as fast as they could until voluntary fatigue. This last stage lasted between 30 and 90 s for all subjects. The protocol is based on a traditionally used incremental *V*O_2peak_ test, previously described in, e.g., Helgerud et al. (2007) and Johansen et al. (2022). However, the last freely chosen all-out phase of the test was new in this study. This phase was added because of the exponential increase in power related to the increase in poling velocity. As the poling ergometer is braked by air resistance from a turbine, the actual brake power increases with velocity^3^. This combined protocol is the result of internal pilot testing aiming to find the protocol best fitted to obtain *V*O_2peak_. The point where the incremental part of the protocol ended and the last phase started was based on evaluation of heart rate (HR) ≥ 90% of HR_max_, a marked increase in ventilation, a respiratory exchange ratio ≥ 1.00, as well as a subjective evaluation of the subjects increasing effort to maintain the pace. At the end of the test, voluntary fatigue, peak heart rate (HR_peak_) ≥ 95% of HR_max_, respiratory exchange ratio (RER), as well as a plateau of the *V*O_2_ curve were used as criteria to evaluate if *V*O_2peak_ was obtained. These criteria have previously been used in, e.g., Helgerud et al. (2007) and Johansen et al. (2022). The mean of the two subsequent highest registered *V*O_2_-values, each representing 20 s intervals by the mixing chamber, was set as *V*O_2peak_. Immediately after the test, a capillary blood sample to measure blood lactate concentration ([La^−^]_b_) was taken. [La^−^]_b_ was measured with a Lactate Scout + (SensLab GmbH, Leipzig, ray Inc., Kyoto, Japan).

A 1-h break was given between the *V*O_2peak_ and the *C* test. The C test was then carried out as a 5-min work period at a break power representing 70–90% of *V*O_2peak_ obtained from the *V*O_2peak_ test. The average of four 20 s measurements between minutes 3 and 4 was used to calculate *C* as *V*O_2_ in mL kg^−1^ w^−1^. Based on the *V*O_2peak_ and C measurements, MAP was calculated as *V*O_2peak_ × *C*^−1^. This equation has previously been validated and explained in Helgerud et al. (2010), and used in, e.g., Støren et al. (2008), Støren et al. (2021) and Johansen et al. (2022).

The subjects were tested in TTE at 130% of MAP immediately after the C test. This test consisted of measurements of time, HR and *V*O_2_ during DP to voluntary exhaustion at the watt representing 130% of MAP. Voluntary exhaustion was defined as the point where the subjects made three or more pole strides below the predetermined watt. [La^−^]_b_ was measured immediately after the test.

The second day of testing consisted of two time trials, 100TT and 800TT. After a 15-min warm up on the ski ergometer, the subjects completed the 100TT as an all-out test. Mean and peak watt, and time performance were measured. After completing the 100TT, the subjects had an active break of 15 min, were they poled at a low intensity of approximately 60% of HR_max_. Before the 800TT started, the outlay watt was set based on the watt and time spent at TTE. From the outlay, the subjects tried to complete the test as fast as possible. HR and *V*O_2_ was measured continuously, with *V*O_2_ measurements from the mixing chamber every 5th second. [La^−^]_b_ was measured immediately after the test. Before the 100TT and 800TT, the subjects had been familiarized with the poling ergometer, by poling at different velocities and intensities during test day one. Prior to the first test in test day one, they also had a 15–30 min familiarization to the equipment. However, no specific familiarization to the specific 100TT or 800TT was performed before the actual tests. The velocity at the TTE-test at 130% MAP in test day one served as starting point from which the subjects could calculate their outlay speed in the 800TT.

MAOD was calculated as the mean difference between *V*O_2_ demand and measured *V*O_2_ in 800TT (Medbo et al. 1988), and expressed as both the product of this difference and time (mL kg^−1^), the difference per minute (mL kg^−1^ min^−1^), as well as relative to *V*O_2peak_ (%*V*O_2peak_). *V*O_2_ demand was calculated as the product of mean race power and the oxygen cost, i.e., *w* × *C* (Billat et al. 2009). For example, a skier had a mean 800TT power of 200*w*, a *C* of 0.300 mL kg^−1^ w^−1^, and a race time of 150 s (2.5 min). *V*O_2_ demand was then 200*w* × 0.300 mL kg^−1^ w^−1^ = 60 mL kg^−1^ min^−1^. If the mean *V*O_2_ during the 800TT was 45 mL kg^−1^ min^−1^, MAOD would be 60–45 = 15 mL kg^−1^ min^−1^, or 15 mL kg^−1^ min^−1^ × 2.5 min = 37.5 mL kg^−1^. APR was calculated as the difference between MANP and MAP and is presented in absolute values (*w*) and also as a percent of MAP (%MAP).

### Statistics

Normality was tested using QQ-plots and Shapiro–Wilk (*p* = 0.10) and found to represent normal distributions for the main variable (800TT). Values were thus expressed descriptively as mean ± standard deviation (SD), as well as the coefficient of variance (%). As parametric statistics were used, the slowest and fastest skiers were divided by above or below mean 800 m time. Differences between the slowest and fastest skiers were analyzed by an independent samples *t* test. The two groups still represented a normal distribution each (Shapiro–Wilk tests with *p* = 0.15 and *p* = 0.50 in the slowest and fastest group, respectively). The slowest and the fastest group had 8 participants (four females) and 10 participants (one female), respectively. All correlations were expressed as the correlation factor *r* from Pearson’s bivariate tests and supplemented with the standard error of the estimate. As a supplement, the correlations were repeated corrected for sex in partial correlations. A *p* value < 0.05 was accepted as statistically significant in all tests. Multiple regressions were performed, with 800TT as the dependent variable. Only the results for 800TT versus MAP × MANP, and 800TT versus MAP × MANP × MAOD were presented. There are three main reasons for why these results were not further discussed. The number of participants were too low for multiple regressions, the level of co-linearity between the independent variables were too high (VIF 4.5–9.3), and the main purpose of the study was to investigate the relationship between the single variables and 800TT. All analyses were performed using the software program Statistical Package for Social Sciences, version 27 (SPSS, IBM, Chicago, IL USA).

## Results

Performance- and physiological results are presented in Table 2, both as all skiers together, and divided by over- and under mean 800TT performance.

**Table 2:** Performance and physiological results (*N* = 18)

	All (*N* = 18)	800TT > 192 s (*N* = 8)	800TT < 192 s (*N* = 10)
*V*O_2peak_			
L min^−1^	3.76 ± 0.82 (21.8)	3.23 ± 0.69 (21.4)	4.20 ± 0.66 (15.7)**
mL kg^−1^ min^−1^	50.5 ± 9.2 (18.2)	44.4 ± 5.5 (12.4)	55.4 ± 8.7 (15.7)**
HR (BPM)	185 ± 11 (5.7)	185 ± 9 (4.9)	185 ± 12 (6.5)
RER (*V*CO_2_/*V*O_2_)	1.15 ± 0.07 (6.1)	1.14 ± 0.08 (7.0)	1.16 ± 0.06 (5.2)
*C*			
mL kg^−1^ w^−1^	0.297 ± 0.105 (35.4)	0.384 ± 0.099 (25.8)	0.229 ± 0.035 (15.3)**
MAP			
*w*	191.4 ± 76.0 (39.7)	122.3 ± 33.9 (27.7)	246.6 ± 48.8 (19.8)**
MANP			
*w*	452.8 ± 133.6 (29.5)	341.4 ± 115.1 (33.7)	541.9 ± 59.7 (11.0)**
APR			
*w*	261.4 ± 76.2 (29,2)	219.1 ± 89.5 (40.8)	295.3 ± 43.4 (14.7)
%MAP	249.0 ± 48.2 (19.4)	279.3 ± 47.5 (17.0)	224.8 ± 34.2 (15.2)*
800 m			
TT (s)	192.2 ± 28.8 (15.0)	217.4 ± 23.2 (10.7)	172.0 ± 11.7 (6.8)**
HR (BPM)	183.0 ± 7.3 (4.0)	183 ± 8 (4.4)	183 ± 7 (3.8)
Mean power (w)	226.1 ± 87.8 (38.8)	147.8 ± 43.3 (29.3)	288.8 ± 57.5 (19.9)**
0.8MAP + 0.2MANP	243.6 ± 85.2 (35.0)	166.1 ± 47.8 (28.8)	305.7 ± 48.1 (15.7)**
%MAP (%)	119.9 ± 11.6 (9.7)	121.2 ± 16.8 (13.9)	117.2 ± 5.4 (4.6)
% MANP (%)	41.5 ± 7.6 (18.3)	36.7 ± 6.2 (16.9)	45.4 ± 6.4 (14.1)*
[La^−^]_b_(mM)	12.1 ± 3.8 (31.4)	11.5 ± 2.5 (21.7)	12.6 ± 4.7 (37.3)
MAOD (mL kg^−1^)	61.2 ± 28.3 (46.2)	73.5 ± 29.6 (40.3)	51.3 ± 25.2 (49.1)
MAOD (mL kg^−1^ min^−1^)	19.0 ± 8.4 (44.2)	20.5 ± 8.2 (40.0)	17.8 ± 8.9 (50.0)
MAOD (%*V*O_2peak_)	125.4 ± 61.9 (49.3)	164.9 ± 56.0 (34.0)	93.9 ± 48.2 (51.3)*
100 m			
TT (s)	20.1 ± 2.5 (12.4)	22.1 ± 2.4 (10.9)	18.5 ± 0.9 (4.9)**
Mean power (w)	370.1 ± 113.4 (30.6)	278.3 ± 96.0 (34.5)	443.5 ± 60.3 (13.6)**
Peak power (w)	452.8 ± 133.6 (29.5)	341.4 ± 115.1 (33.7)	541.9 ± 59.7 (11.0)**
%MAP (%)	249.0 ± 48.2 (19.4)	279.3 ± 47.5 (17.0)	224.8 ± 34.2 (15.2)*
TTE at 130% MAP			
s	104.9 ± 62.0 (59.1)	126.0 ± 85.5 (67.9)	88.1 ± 29.4 (33.1)
HR (BPM)	184.0 ± 7.4 (4.0)	183 ± 9 (4.9)	184 ± 6 (3.3)
[La^−^]_b_	10.5 ± 3.2 (30.5)	10.6 ± 2.2 (20.8)	10.5 ± 3.9 (37.1)

Values are mean ± standard deviation, with coefficient of variance in percent in parenthesis

*VO*_*2peak*_ peak oxygen consumption, *C* oxygen cost of double poling, *HR* heart rate, *BPM* beats per minute, *RER* respiratory exchange ratio, *w* watts, *MAP* maximal aerobic power (*V*O_2peak_/*C*), *MANP* maximal anaerobic power, *ASR* anaerobic sprint reserve, *[La*^*−*^*]*_*b*_ blood lactate concentration in millimole per liter (mM), *TT* time results in the 800 m or the 100 m, *s* seconds, *MAOD* mean accumulated oxygen deficit, *TTE at 130% MAP* time to exhaustion at 130% of MAP

*p < 0.05 different from > 192 s

**p < 0.01 different from > 192 s

**Fig. 1:**
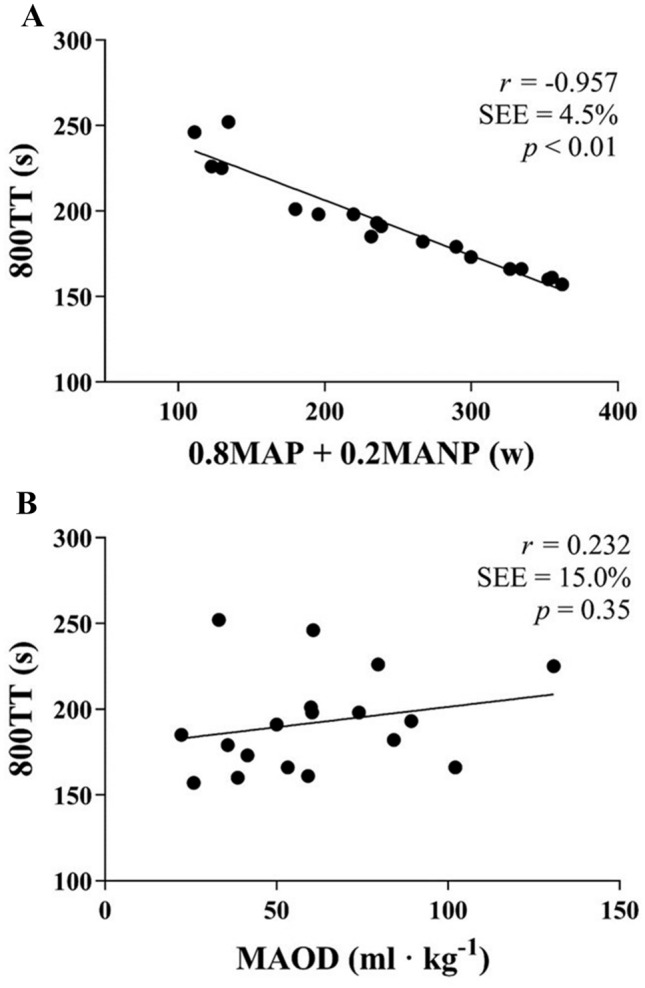
Associations between 0.8MAP + 0.2MANP (**A**), MAOD (**B**), and time performance in 800 m double poling. 0.8MAP + 0.2MANP is presented as watt (w), MAOD is presented as milliliters per kilogram body mass (mL kg^−1^), and time performance in 800 m double poling (800TT) is presented as seconds (s)

Correlations with time performance and with MAOD are presented in Tables 3 and 4 and Fig. 1.

**Table 3:** Correlations with time performances (*N* = 18)

	800TT (s)	100TT (s)	TTE 130% MAP (s)
*V*O_2peak_			
L min^−1^	− 0.849 (8.2)**	− 0.864 (6.4)**	− 0.262 (58.8)
mL kg^−1^ min^−1^	− 0.733 (10.5)**	− 0.613 (10.0)**	− 0.265 (58.7)
*C*			
mL kg^−1^ w^−1^	0.863 (7.8)**	0.870 (6.2)**	0.698 (43,6)**
MAP			
*w*	− 0.936 (6.0)**	− 0.858 (6.5)**	− 0.497 (52.9)*
MANP			
*w*	− 0.922 (6.0)**	− 0.982 (2.3)**	− 0.413 (55.5)
APR			
*w*	− 0.682 (11.3)**	− 0.866 (6.3)**	− 0.229 (59.3)
%MAP	0.513 (13.3)*	0.275 (12.1)	0.538 (51.3)*
*800 m*			
TT (s)		0.938 (5.4)**	0.428 (55.1)
0.8MAP + 0.2MANP	− 0.957 (4.5)**	− 0.920 (4.9)**	− 0.484 (53.3)*
[La^−^]_b_ (mM)	− 0.074 (15.4)	− 0.128 (12.5)	− 0.309 (57.9)
MAOD (mL kg^−1^)	0.232 (15.0)	0.153 (12.5)	0.559 (50.5)*
MAOD (mL kg^−1^ min^−1^)	− 0.049 (15.4)	− 0.129 (12.5)	0.371 (56.6)
100 m			
TT (s)	0.938 (5.4)**		0.439 (54.7)
Peak power (w)	− 0.922 (6.0)**	− 0.982 (2.3)**	− 0.413 (55.5)
TTE at 130% MAP			
s	0.428 (14.0)	0.439 (11.3)	
[La^−^]_b_ (mM)	0.001 (15.4)	− 0.186 (12.4)	0.009 (60.9)

Values are the correlation coefficient *r*, with the standard error of estimate in parenthesis

*VO*_*2peak*_ peak oxygen consumption, *C* oxygen cost of double poling, *HR* heart rate, *BPM* beats per minute, *RER* respiratory exchange ratio, *w* watts, *MAP* maximal aerobic power (*V*O_2peak_/*C*). *MANP* maximal anaerobic power, *ASR* anaerobic sprint reserve, *[La*^*−*^*]*_*b*_ blood lactate concentration in millimole per liter (mM), *TT* time results in the 800 m or the 100 m, *s* seconds, *MAOD* mean accumulated oxygen deficit, *TTE at 130% MAP* time to exhaustion at 130% of MAP

*p < 0.05 significant correlation

**p < 0.05 significant correlation

**Table 4:** Correlations with MAOD (*N* = 18)

	MAOD (mL kg^−1^)	MAOD (mL kg^−1^ min^−1^)	MAOD (%*V*O_2peak_)
800 m			
TT (s)	0.232 (46.4)	− 0.049 (45.8)	0.457 (45.4)
[La^−^]_b_ (mM)	0.004 (47.7)	0.073 (45.8)	0.053 (50.9)
TTE at 130% MAP			
s	0.559 (39.5)*	0.371 (42.6)	0.620 (39.9)**
[La^−^]_b_ (mM)	0.151 (47.1)	0.192 (44.7)	0.198 (49.9)
MAP			
*w*	− 0.386 (44.0)	− 0.110 (45.3)	− 0.621 (39.9)**
MANP			
*w*	− 0.358 (47.2)	− 0.102 (45.3)	− 0.545 (47.6)*
APR			
*w*	0.131 (47.2)	0.154 (42.6)	− 0.002 (50.9)
%MAP	0.648 (36.3)**	0.498 (39.5)*	0.782 (31.7)**

Values are the correlation coefficient *r*, with the standard error of estimate in parenthesis

*VO*_*2peak*_ peak oxygen consumption, *C* oxygen cost of double-poling, *HR* heart rate, *BPM* beats per minute, *RER* respiratory exchange ratio, *w* watts, *MAP* maximal aerobic power (*V*O_2peak_/*C*), *MANP* maximal anaerobic power, *ASR* anaerobic sprint reserve, *[La*^*−*^*]*_*b*_ blood lactate concentration in millimole per liter (mM), *TT* time results in the 800 m or the 100 m, *s* seconds, *MAOD* mean accumulated oxygen deficit, *TTE at 130% MAP* time to exhaustion at 130% of MAP

*p < 0.05 significant correlation

**p < 0.01 significant correlation

A multiple linear regression between 800TT and MAP × MANP resulted in *r* = 0.96 (*p* < 0.01, SEE = 4.5). The variation inflation factor (VIF) between MAP and MANP against 800TT was 4.38. This indicates collinearity between MAP and MANP regarding correlations with 800TT. It further indicates that the fastest (MANP) skiers also had the highest MAP. A correlation between MAP and MANP exhibited an *r*-value of 0.88 (*p* < 0.01, SEE = 19.6%).

A multiple linear regression between 800TT and MAP × MANP × MAOD resulted in* r* = 0.97 (*p* < 0.01, SEE = 4.3). The variation inflation factor (VIF) between MAP and MANP and MAOD against 800TT was 9.28. This indicates collinearity also between MAP, MANP and MAOD regarding correlations with 800TT.

Since the data material consisted of 13 males and 5 females, correlations were also corrected for sex (supplementary tables s1 and s2). Main findings were not statistically different, corrected, or uncorrected for sex.

## Discussion

The main findings in the present study were that MAP and MANP were strongly correlated with time performance in 800TT, and that neither TTE nor MAOD had any correlations with 800TT performance. MAOD correlated moderately with TTE and with APR.

Mean time performance in the 800 m (192 s) was somewhat shorter than the 207 s found in Andersson et al. (2010) and 213 s in Sandbakk et al. (2011c). The participants represented a heterogeneous cohort shown by a CV of 15% in the 800TT, and as much as 39% in mean power in the 800TT. Since MAP and MANP were found to represent co-linearity (VIF = 4.38), a better way to express the relationship with 800TT by MAP and MANP was to use the equation 0.8MAP + 0.2MANP. The equation was based on the mean intensity of 120% of MAP in the 800TT. This equation correlated strongly (*r* = 0.957, *p* < 0.01, SEE = 4.4%) with 800TT. The mean 80%/20% ratio between MAP and MANP is in accordance with the same ratio in aerobic/anaerobic metabolic demand previously shown in Losnegard et al. (2012) and Andersson et al. (2017). When comparing the fastest skiers with the slowest skiers, the fastest skiers had higher MAP and MANP, but not significantly different TTE or MAOD. The exception is when MAOD was expressed as %*V*O_2peak_, where the slowest skiers had the highest MAOD. The relationship between MAP and 800TT is in accordance with previous studies on sprint cross-country skiing (Stöggl et al. 2007; Sandbakk et al. 2011a, b; Losnegard et al. 2012; Carlsson et al. 2016; Andersson et al. 2017; Hébert-Losier et al. 2017). The lack of relationship between MAOD and 800TT is in contrast to results from Losnegard et al. (2012, 2013) in sprint skiing, and Ramsbottom et al. (1994) in running, finding that the fastest runners had the highest MAOD. However, the present results are in accordance with results from Craig and Morgan (1998) in running, finding no significant relationship between MAOD and 800TT.

There was not an even distribution of males and females in the present study (13 males and 5 females). In Sollie and Losnegard (2022), distance covered in a 3 min roller skiing TT was approximately 20% longer in males than in female elite skiers. The sex difference in *V*O_2peak_ was the same as the difference in TT in their study. The Sollie and Losnegard (2022) study is an example of sex differences being attributed to differences in physiological variables. We, therefore, argue that when comparing time performance with physiological variables, this can be done independent of sex. The low number of females would also make it irrelevant to perform separate correlations for each sex. However, in order to see a possible impact of sex on the results, partial correlations corrected for sex were performed (tables s1 and s2). Main findings were not different, corrected, or uncorrected for sex.

Neither MAOD nor TTE correlated with 800TT in the present study. MAOD, both expressed as mL kg^−1^, and %*V*O_2peak_, correlated moderately with TTE. It could be argued that by measuring MAOD in the actual 800TT, the best skiers would be underestimated since they performed the shortest time. To account for this, MAOD was also presented per min. However, MAOD did still not correlate with 800TT. It could also be argued that MAOD would be overestimated among the fastest skiers and underestimated among the slowest because of large and small absolute numbers, respectively. To account for this, MAOD was presented relative to *V*O_2peak_ as well. There was still no correlation between MAOD and 800TT. The lack of relationship between MAOD and 800TT may indicate that anaerobic capacity measured as MAOD is not relevant as an endurance measure. MAOD seems to be a set volume for each individual, as shown in Hill and Vingren (2011) where MAOD was approximately the same in all out exercise in 3, 5, or 7 min. In the present study, MAOD correlated with TTE at 130% MAP. In addition, APR correlated with MAOD in the present study. The present results thus support the model proposed by Billat et al. (2009) on middle distance running performance, that any instant running speed should be controlled by the prevailing anaerobic store remaining. As those with the highest MANP in the present study would perform both 800TT and TTE at a lower per cent of MANP, the set anaerobic store would be portioned out to last longer. As proposed in Støren et al. (2021), a possible consequence of this is that MANP sets the potential for anaerobic capacity, and that both MAOD and TTE are simply measures of this potential portioned out.

APR was presented both in absolute values and relative to MAP in the present study. We argue that the latter expression of APR is the most accurate in a heterogeneous performance group. Absolute values would overestimate APR in those with the highest MAP and, or MANP, while underestimating those with low MAP or MANP. The importance of this is clearly shown in Table 3, where there is a moderate negative correlation between 800TT and APR (*w*), and a moderate positive correlation between 800TT and APR (%MAP). The suggestion made in Støren et al. (2021), that APR relative to MAP theoretically should be a significant determinant of MAOD, was confirmed in the present study with a strong correlation between APR (%MAP) and MAOD (%*V*O_2peak_). There was also a moderate correlation between APR (%MAP) and TTE in the present study. Although the correlation was moderate, it is in accordance with the results in running from Blondel et al. (2001) and Støren et al (2021). The relationships between APR (%MAP) and MAOD and TTE thus points at the same suggestion made in Støren et al. (2021) in running, that MANP sets the potential for anaerobic capacity, and that those who can portion this out the best due to a high APR can complete the longest time at a given supramaximal intensity relative to MAP.

The equivocal findings in relationships between MAOD and ski sprint or middle-distance running time performance in the previous studies (Ramsbottom et al. 1994; Craig and Morgan 1998; Losnegard et al. 2012, 2013) could be related to APR. We hypothesize, based on the present results, that athletes with a high APR caused by a high MANP may have a negative relationship between MAOD and time spent. We also hypothesize that athletes with a high APR caused by a low MAP may have a positive relationship between MAOD and time spent. The heterogeneity in 800TT, MAP, MANP and APR results in the present study may, therefore, be a reason for the lack of relationship between MAOD and 800TT. However, neither of the previous studies measured both MAP, MANP, APR, MAOD and time performance in the same study. This hypothesis can, therefore, not yet be confirmed or rejected.

### Practical implications, limitations, and future perspectives

Addressing physiological variables with the same denotations as the performance variables, i.e., as velocity or power such as MAP and MANP, may prove a useful tool in effectively identifying and quantifying variables of importance. The present results points at MAP and MANP, but not MAOD or TTE as such variables regarding 800TT in DP. If the relationships, and the lack of relationships presented in the present study should represent causality, it would have certain consequences for training strategies. One strategy would be to improve MANP in order to pole faster at the same submaximal percentage of maximal speed or watt. Another strategy would be to improve MAP, in order to be able to pole faster at the same supramaximal percentage of maximal aerobic speed or watt. In order to improve coordination, selective activation of motor units, neural firing rate, and thus the maximal power output (Škarabot et al. 2021), a combination of maximal strength training and sprint training could prove useful. Maximal strength training has been shown to both improve C in running (Støren et al. 2008) and in DP (Østerås et al. 2002). Maximal strength training also has the potential to improve sprint performance, as has short sprint training shown by Rumpf et al. (2016) in running. However, few studies have addressed the effect of sprint training or maximal strength training on short-sprint skiing performance (Losnegard et al. 2011; Skattebo et al. 2016; Carlsson et al. 2017), and with equivocal effects. In addition to maximal strength training to improve *C*, high intensity aerobic interval training has been shown to improve *V*O_2peak_, (Johansen et al. 2020). An increase in *V*O_2peak_ in DP could result from either an increase in the utilization of *V*O_2max_ in DP (Johansen et al. 2020), or an increase in *V*O_2max_ due to an improvement of, e.g., stroke volume (Helgerud et al. 2007), or a combination of both.

The present data were obtained from DP on a ski ergometer, and caution should be taken in generalizing these results on to ski-racing on snow and with use of several different skiing techniques. On the other hand, to investigate the relationships between aerobic and anaerobic power, anaerobic capacity, APR, and time performance in DP in a ski ergometer do not need to be representative for snow skiing performance to be of interest. The methodology in assessing possible correlations between these variables should still be valid. If for instance a skier has better C on snow than in the ski ergometer, it would simply mean a better MAP, and quite possibly a better TT. The present results give a base for future intervention studies to investigate the causality of the correlations. In order to investigate causality further, we propose to perform short-sprint or maximal strength training interventions aiming to improve MANP, and high intensity aerobic interval training in DP in order to improve MAP, and to investigate the effects of an improved MANP or MAP on ski sprint TT performance, MAOD, TTE and APR.

## Conclusions

800TT in ergometer double poling was determined by MAP and MANP, but not by TTE or MAOD. This may indicate little or no impact from anaerobic endurance capacity in an all-out event lasting approximately 3 min.

## Supplementary Information

Below is the link to the electronic supplementary material.

Supplementary file1 (PDF 48 KB)

Supplementary file2 (PDF 43 KB)

## Data Availability

The data sets generated during and/or analyzed during the current study are available from the corresponding author on reasonable request.

## Abbreviations

APR: Anaerobic power reserve
ASR: Anaerobic sprint reserve
BW: Body weight
*C*: Oxygen cost of double poling
DP: Double poling
HR: Heart rate
HR_max_: Max heart rate
HR_peak_: Peak heart rate
[La^−^]_b_: Blood lactate concentration
MANP: Maximal anaerobic power
MANS: Maximal anaerobic speed
MAOD: Maximal accumulated oxygen deficiency
MAP: Maximal aerobic power
MAS: Maximal aerobic speed
RER: Respiratory exchange ratio
TT: Time trial
TTE: Time to exhaustion
*V*O_2max_: Maximal oxygen uptake
*V*O_2peak_: Peak oxygen uptake
w: Watt
