# Supplementary material for: Maximal aerobic and anaerobic power and time performance in 800 m double poling ergometer
Source: Eur J Appl Physiol. 2023 Feb 7;123(6):1199–208. doi: 10.1007/s00421-023-05149-9 (PMC10192160; doi:10.1007/s00421-023-05149-9)
Supplement: Supplementary file 1 — Supplementary file1 (PDF 48 KB) [file 421_2023_5149_MOESM1_ESM.pdf]

Table s1. Correlations with time performances, corrected for sex (N=18, 13 males and 5 females)

	800TT (s)	100TT (s)	TTE 130% MAP (s)
**VO_{2peak}**			
$L·min^{-1}$	-0.748**	-0.748**	-0.290
$mL·kg^{-1}·min^{-1}$	-0.617**	-0.420	-0.265
**C**			
$mL·kg^{-1}·w^{-1}$	0.859**	0.894**	0.727**
**MAP**			
w	-0.923**	-0.822**	-0.528*
**MANP**			
w	-0.873**	-0.968**	-0.489*
**APR**			
w	-0.459	-0.752**	-0.245
%MAP	0.710**	0.446	0.548*
**800m**			
TT (s)		0.901**	0.482
0.8MAP+0.2MANP	-0.938**	-0.888**	-0.533*
$[La^-]_b$(mM)	-0.110	-0.192	-0.311
MAOD ($mL·kg^{-1}$)	0.402	0.334	0.576*
MAOD ($mL·kg^{-1}·min^{-1}$)	-0.168	-0.094	0.412
**100m**			
TT (s)	0.901**		0.525*
Peak power (w)	-0.873**	-0.968**	-0.489*
**TTE at 130% MAP**			
s	0.482	0.525*	
$[La^-]_b$(mM)	0.148	-0.082	0.023

Values are the correlation coefficient r. VO_{2peak}, peak oxygen consumption. C, oxygen cost of double poling. HR, heart rate. BPM, beats per minute. RER, respiratory exchange ratio. W, watts. MAP, maximal aerobic power (VO_{2peak} / C). MANP, maximal anaerobic power. ASR, anaerobic sprint reserve. $[La^-]_b$, blood lactate concentration in millimole·L^{-1} (mM). TT, time results in the 800m or the 100m. s, seconds. MAOD, mean accumulated oxygen deficit. TTE at 130% MAP, time to exhaustion at 130 per cent of MAP.

*$p<0.05$ significant correlation

** $p<0.05$ significant correlation
