# Supplementary material for: Maximal aerobic and anaerobic power and time performance in 800 m double poling ergometer
Source: Eur J Appl Physiol. 2023 Feb 7;123(6):1199–208. doi: 10.1007/s00421-023-05149-9 (PMC10192160; doi:10.1007/s00421-023-05149-9)
Supplement: Supplementary file 2 — Supplementary file2 (PDF 43 KB) [file 421_2023_5149_MOESM2_ESM.pdf]

**Table s2. Correlations with MAOD, corrected for sex (N=18, 13 males and 5 females)**

| | MAOD ($mL\cdot kg^{-1}$) | MAOD ($mL\cdot kg^{-1}\cdot min^{-1}$) | MAOD (%$VO_{2peak}$) |
|---|---|---|---|
| **800m** | | | |
| TT (s) | 0.402 | 0.168 | 0.532* |
| $[La^-]_b$ | -0.001 | 0.182 | 0.052 |
| **TTE at 130% MAP** | | | |
| s | 0.576 * | 0.412 | 0.618** |
| $[La^-]_b$(mM) | 0.129 | 0.146 | 0.213 |
| **MAP** | | | |
| w | -0.523* | -0.300 | -679** |
| **MANP** | | | |
| w | -0.322 | -0.078 | -0425 |
| **APR** | | | |
| w | 0.050 | 0.221 | 0.052 |
| %MAP | 0.645 ** | 0.497 * | 0.790** |

Values are the correlation coefficient r. $VO_{2peak}$, peak oxygen consumption. C, oxygen cost of double poling. HR, heart rate. BPM, beats per minute. RER, respiratory exchange ratio. W, watts. MAP, maximal aerobic power ($VO_{2peak}$ / C). MANP, maximal anaerobic power. ASR, anaerobic sprint reserve. $[La^-]_b$, blood lactate concentration in millimole$\cdot L^{-1}$ (mM). TT, time results in the 800m or the 100m. s, seconds. MAOD, mean accumulated oxygen deficit. TTE at 130% MAP, time to exhaustion at 130 per cent of MAP.

*p<0.05 significant correlation

** p<0.01 significant correlation
